# Impact of local thermal stimulation on the correlation between oxygen saturation and speed-resolved blood perfusion

**DOI:** 10.1038/s41598-019-57067-6

**Published:** 2020-01-13

**Authors:** Guangjun Wang, Shuyong Jia, Mi Liu, Xiaojing Song, Hongyan Li, Xiaorong Chang, Weibo Zhang

**Affiliations:** 1 Institute of Acupuncture and Moxibustion, China Academy of Chinese Medical Sciences, Beijing, China; 2 Acupuncture and Tuina School, Hunan University of Chinese Medicine, Changsha, China

**Keywords:** Preclinical research, Translational research

## Abstract

The physiologically important relationship between oxygen saturation and blood flow is not entirely understood, particularly with regard to the multiple velocity components of flow and temperature. While our previous studies used classic laser Doppler flowmetry combined with an enhanced perfusion probe to assess local blood flow following thermal stimulation, oxygen saturation signals were not assessed. Thus, the current study used multiscale entropy (MSE) and multiscale fuzzy entropy (MFE) to measure the complexity of oxygen saturation signals following thermal stimulation in healthy subjects. The results indicate that thermal stimulation increases oxygen saturation and affects the measured signal complexity in a temperature-dependent fashion. Furthermore, stimulus temperature not only affects the correlation between speed-resolved blood perfusion and oxygen saturation, but also the correlation between the complexity area indices (CAI) of the two signals. These results reflect the complexity of local regulation and adaptation processes in response to stimuli at different temperatures.

## Introduction

While the classic technique laser Doppler flowmetry (LDF) has been frequently used to measure blood flow in tissues, it cannot clearly differentiate between different vascular compartments. Recently, a multiparameter model based on the Monte Carlo algorithm has made it possible to more precisely resolve the different velocity components in microcirculatory perfusion^1–4^. By integrating diffuse reflectance spectroscopy (DRS) and laser Doppler flowmetry (LDF) into a single fiber-optic probe, the Enhanced Perfusion and Oxygen Saturation (EPOS) system can measure blood flow and blood oxygen saturation simultaneously^5^. This new method may provide further insight into vascular dysfunction at both local and systemic levels^6,7^.

Oxygen saturation arises from a dynamic balance between O_2_ supply and consumption in capillary, arteriolar, and venular beds and it is generally believed that local oxygen saturation is closely related to blood perfusion^8^. Previous studies have demonstrated an exponential correlation between oxygen saturation and blood flow. When the speed of blood flow increases, the capillary transit time decreases, resulting in an instantaneous decrease in oxygen extraction and an increase in the overall level of oxygen saturation assuming oxygen consumption remains constant^9^. However, while blood flow can be resolved into different components according to speed, the relationship between oxygen saturation and speed-resolved blood perfusion is still unclear.

Building upon our previous study^10^, which measured speed-resolved blood perfusion using the EPOS system, the present study combined these measurements with a simultaneous analysis of the changes in the complexity of oxygen saturation signals in response to thermal stimuli to clarify the relationship between oxygen saturation and speed-resolved blood perfusion. As the regulation of the circulatory system is known to be a nonlinear process^11^, sample entropy and multiscale entropy (MSE) have been used to evaluate the skin blood flow oscillations^12–14^.

Generally, human physiological signals, including EEG and ECG, are inherently complex^15^, which refers to the nonrandom fluctuations in the irregular dynamics of signals^16^. MSE can quantify the irregularity of a signal on multiple time scales^17^ and which has been widely used to analyze the complexity of physiological signals^18^. The signal with larger entropy is considered to be more complex which means the signal contains richness information. Multiscale fuzzy entropy (MFE), as an enhanced MSE method, is also an effective method for evaluating the complexity of time series. Relevant research shows that MFE has a more significant correlation with MSE than other multiscale entropies^19^.

In our previous study, both MSE and MFE were used to assess the complexity of thermal stimulation on blood perfusion in the skin^10,20^. Compared with the calculation procedure of MSE, MFE replaces the Heaviside function with fuzzy membership function, which makes MFE less dependent on data length and more resistance to noise. In the current study, both MSE and MFE analysis were used because of two considerations: First, although MFE and MSE have similar meaning, and MFE as an enhanced MSE method, but after all, they use different functions in the calculation process, and get different types of entropy. Calculating MSE and MFE at the same time can make the conclusion more solid. Second, due to the 3 Hz sampling rate, the data length in current study is relatively short than previous study. MFE and MSE have different sensitivity to data length and we also want to explore whether the MSE and MFE are different under the condition of such data length.

Especially in previous study, both the different velocity components of blood flow and its complexity changes resulting from different temperature stimulation were analyzed. However, the relationship between oxygen saturation and speed-resolved blood perfusion were not analyzed. We hypothesize that thermal stimulation not only alter the relationship between signals of local oxygen saturation and velocity components, but also change the relationship of complexity between two kinds of signals in a temperature-dependent manner.

## Results

**Figure 1:**
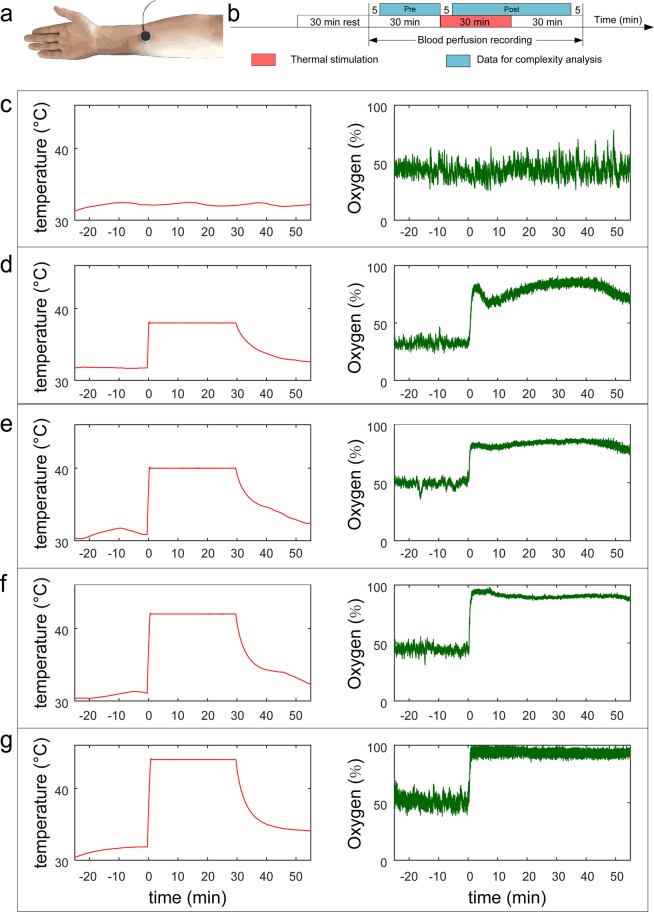
Experiment design and case subject’s raw data. (**a**) Thermal stimulation point. The probe was located on the forearm midline, five twelfths of the line between wrist and elbow. (**b**) Experiment design. From (**c**) to (**g**) are temperature (left) and oxygen saturation signals (right). (**c**) blank control. (**d**) 38 °C. (**e**) 40 °C. (**f)** 42 °C. (**g**) 44 °C.

A total of 60 subjects were recruited in the current study. The results of 59 individuals are reported here (thermal stimulation group, TS, N = 29; blank control group, BC, N = 30). One subject’s oxygen saturation data could not be imported into Matlab software and was excluded from statistical analyses. In the TS group, the order of thermal stimulations was randomly generated for each subject. The different conditions of thermal stimulation and oxygen saturation signals are shown in Fig. 1.

### Oxygen saturation

**Figure 2:**
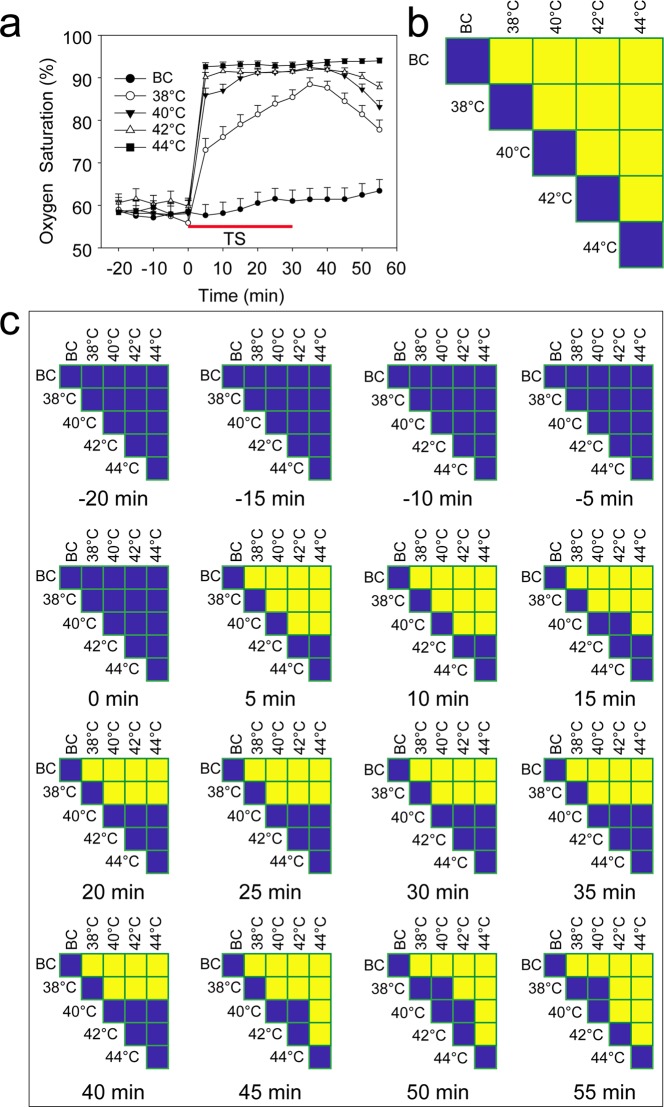
Oxygen saturation at the stimulation point. (**a**) Oxygen saturation signals. (**b**) Design of the comparison. (**c**) Results of the comparison. (*F*_(4,141)_ = 32.63, *P* < 0.0001). Yellow indicates P < 0.05, and blue indicates P > 0.05. BC, blank control; TS, thermal stimulation. Data are presented as the mean ± SE.

The average responses of oxygen saturation to thermal stimulation at the different temperatures over time is shown in Fig. 2. Although the strongest increase generally occurred within 5 min of the start of thermal stimulation, the responses as a whole appear to be temperature dependent. In the case of stimulation at 38 °C, oxygen saturation increased throughout the stimulation period and began to decrease 5 min after the stimulation ended. In contrast, following stimulation at 40 °C, maximum oxygen saturation was reached after 15 min. In response to stimulation at 42 and 44 °C, maximum oxygen saturation was attained even more rapidly (i.e., within 5 min of the start of the stimulation). For the 40, 42, and 44 °C thermal stimulations, the maximal oxygen saturation persisted for at least 30 min, extending to approximately 15 min after the termination of stimulation. For all the temperatures investigated below 44 °C, a decay in the oxygen saturation level occurred in a temperature-dependent manner after the end of each stimulation. No such decay was observed in the case of stimulation at 44 °C over the entire measurement period (Fig. 2a).

### Complexity calculation

**Figure 3:**
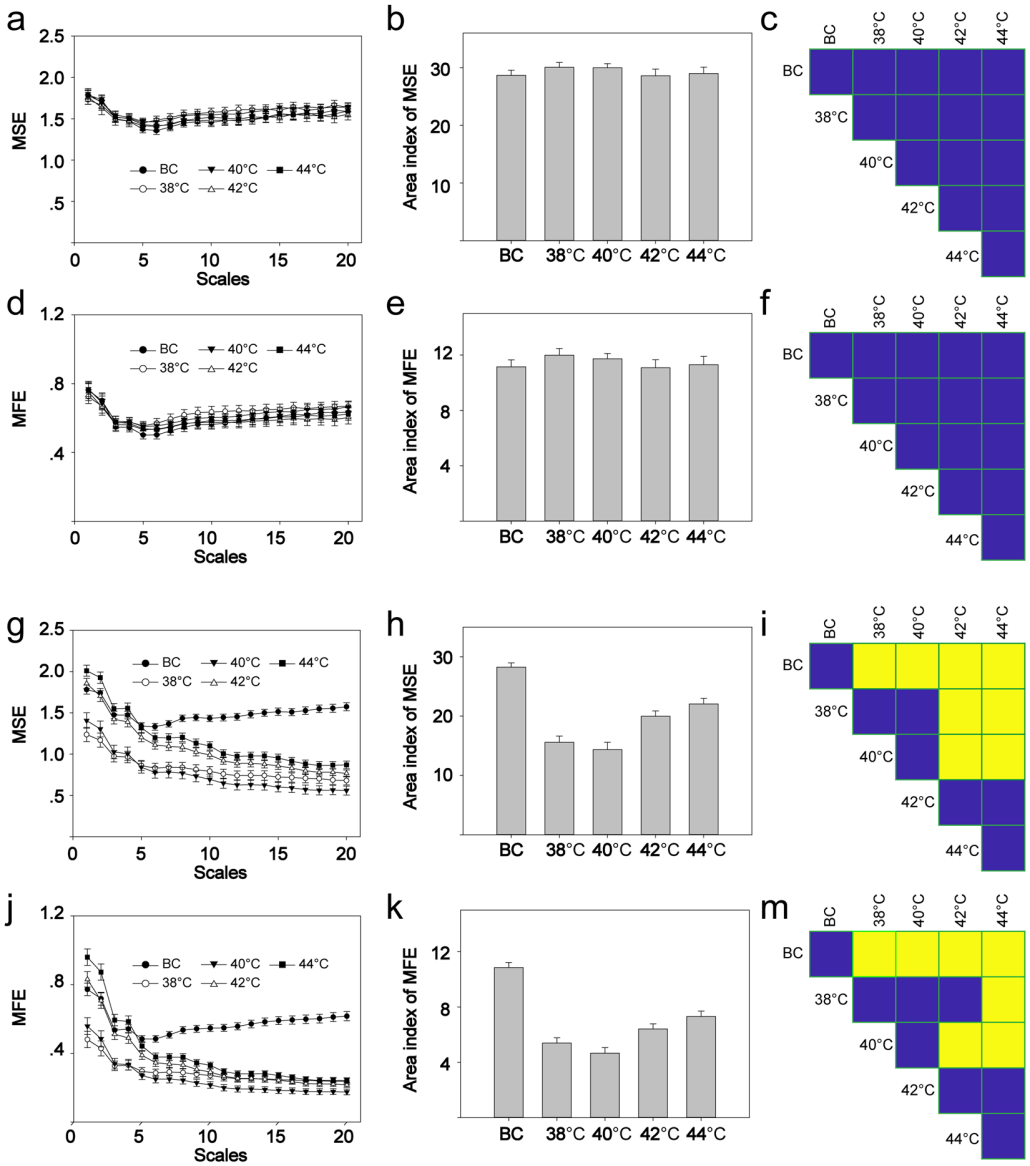
The complexity of oxygen saturation signals. The left column is the curve of MSE/MFE. The middle column is the CAI. The right column is the multiple comparisons. (**a**) MSE before stimulation. (**b**)MSE CAI before stimulation. (**c**) Comparison of MSE CAI before stimulation, *F*_(4,141)_ = 0.5776, *P* = 0.6794. (**d**) MFE before stimulation. (**e**) MFE CAI before stimulation. (**f**) Comparison of MFE CAI before stimulation, *F*_(4,141)_ = 0.5756, *P* = 0.6808. (**g**) MSE after stimulation. (**h**) MSE CAI after stimulation. (**i**) Comparison of MSE CAI after stimulation, *F*_(4,141)_ = 32.38, *P* < 0.01. (**j**) MFE after stimulation. (**k**) MFE CAI after stimulation. (**m**) Comparison of MFE CAI after stimulation, *F*_(4,141)_ = 39.64, *P* < 0.01. Yellow indicates P < 0.05, and blue indicates P > 0.05. MSE, multiscale entropy; CAI, complexity area index; MFE, multiscale fuzzy entropy. BC, blank control. Data are presented as the mean ± SE.

Considering that MFE has been shown to be more closely correlated with MSE than with other multiscale entropy measures^19^, both MSE and MFE were analyzed in the current study. To determine the local oxygen saturation following thermal stimuli, the complexity of the oxygen saturation signals was analyzed using MSE and MFE. In the resting state, neither the MSE nor the MFE differed significantly between the different stimulation groups (Fig. 3a–f). In the case of MSE, stimulation at 38 or 40 °C significantly reduced the complexity area index (CAI). As the temperature of the stimuli increased, the area index also increased. However, the complexity area index at temperatures greater than 42 or 44 °C was still smaller than that of the blank control group (Fig. 3g–i). The results for MFE (Fig. 3j–m) were similar to those of MSE. The relationship of CAI between MFE and MSE is shown in Fig. S1.

**Figure 4:**
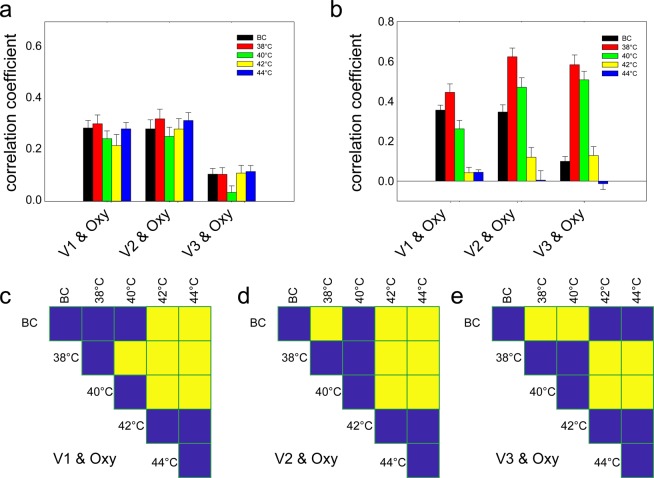
Relationship between speed-resolved perfusion and oxygen saturation signals. (**a**) Before thermal stimulation, V1 & oxygen, *F*_(4,141)_ = 1.245, *P* = 0.34; V2 & oxygen *F*_(4,141)_ = 0.540, *P* = 0.71; V3 & oxygen *F*_(4,141)_ = 1.727, *P* = 0.15; (**b**) After stimulation, V1 & oxygen, *F*_(4,141)_ = 30.923, *P* < 0.001; V2 & oxygen, *F*_(4,141)_ = 29.146, *P* < 0.001; V3 & oxygen, *F*_(4,141)_ = 46.659, *P* < 0.001; From (**c**) to (**d**) are multi comparison after stimulation. (**c**) V1. (**d**) V2. (**e**) V3. Yellow indicates P < 0.05, and blue indicates P > 0.05. V1: low speed component of blood perfusion, <1 mm/s; V2: mid-speed component of blood perfusion, 1–10 mm/s; V3: high speed component of blood perfusion, >10 mm/s; BC, blank control; Oxy, oxygen saturation. Data are presented as the mean ± SE.

We combined the aforementioned analysis of the complexity of local oxygen saturation signals with an analysis of the complexity of speed-resolved blood perfusion^10^. The velocity components V1, V2, and V3 of speed-resolved blood perfusion were computed as described in reference^10^. The comparison of these velocity components with the oxygen-saturation time series was quantified in terms of Spearman’s correlation coefficient, both before (Fig. 4a) and after (Fig. 4b) the application of thermal stimulation.

While there were no significant differences in the results obtained before the start of thermal stimulation (Fig. 4a), there was a significant difference after thermal stimulation (Fig. 4b). For the V1 speed-resolved component, stimulation at 38 °C increased the correlation coefficient with oxygen saturation, while the decrease in the correlation coefficient in response to stimulation at 40 °C was not significantly different than that of the BC group. Moreover, stimulation at 42 and 44 °C significantly reduced the correlation coefficient (Fig. 4b,c). For the V2 speed-resolved component, stimulation at 38 °C increased the correlation coefficient significantly in comparison to the BC group. For the V2 speed-resolved component at stimulation temperatures of 42 and 44 °C, thermal stimulation resulted in a significant reduction in the correlation coefficient (Fig. 4b,d). As for the V3 speed-resolved component, stimulation at both 38 and 40 °C resulted in significant increases in the correlation coefficients in comparison to those of the BC group, while stimulation at 42 and 44 °C resulted in insignificant decreases in the correlation coefficients in comparison to those of the BC group (Fig. 4b,e).

**Figure 5:**
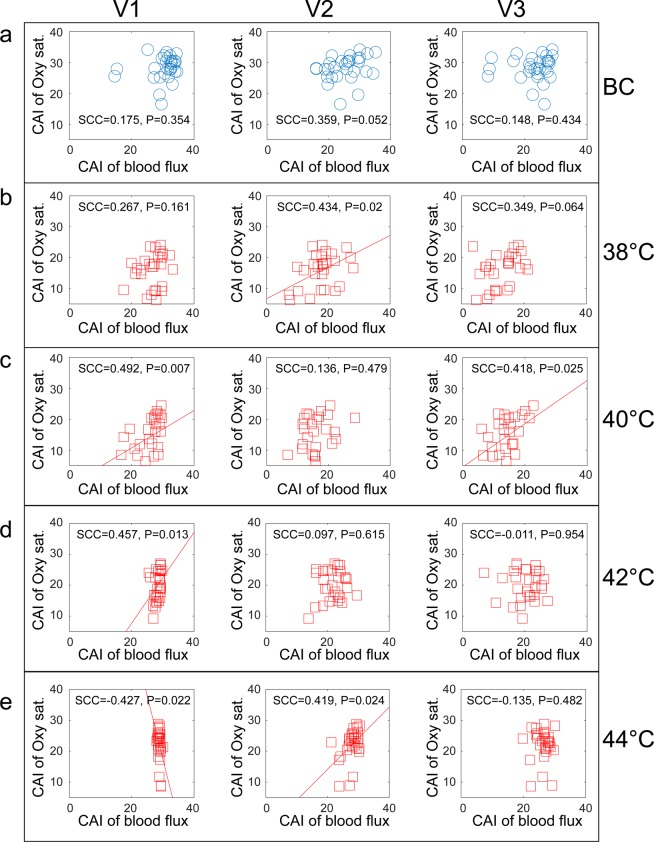
Spearman correlation of MSE CAI between the speed-resolved blood perfusion and oxygen saturation signals. The columns correspond to the three velocity components V1, V2, and V3, and the rows to the stimulation protocol: (**a**) background control, and thermal stimulation at (**b**) 38 °C, (**c**) 40 °C, (**d**) 42 °C, and (**e**) 44 °C. SCC, Spearman’s correlation coefficient; CAI, complexity area index. Oxy sat., oxygen saturation.

The correlation analysis was repeated and applied to the CAI values, which were calculated from the oxygen-saturation and blood-perfusion data. There is no apparent correlation between the MSE-based CAI values derived from the oxygen-saturation data or from any of the blood-velocity components prior to thermal stimulation (Fig. 5a). However, there was a weak correlation between V2 speed-resolved blood perfusion and oxygen saturation in response to thermal stimulation at 38 °C, (Fig. 5b). In response to stimulation at 40 °C, both low-speed components and high-speed components (Fig. 5c) were correlated with oxygen saturation. At 42 °C stimulation, the only correlation was between low-speed components and oxygen saturation (Fig. 5d). Finally, in response to stimulation at 44 °C, there was a negative correlation between oxygen saturation and low-speed components (Fig. 5e), while there was a positive correlation between medium-speed components and oxygen saturation (Fig. 5e). The results of MFE (Fig. S2) are consistent with those of MSE.

## Discussion

Our results highlight the dependence of local oxygen saturation on stimulus temperature by considering the correlation between oxygen saturation and speed-resolved blood perfusion in response to thermal stimulation. When stimulated at 38 or 40 °C, the correlation of the two signals increased to various extents, while when stimulated at 42 and 44 °C, the correlation of the two signals decreased significantly. However, from the point of view of complexity, the correlation changes caused by different temperature stimuli are quite different from the similarity of signals in the time domain. According to our results, the correlation between the oxygen saturation and blood flow signals is enhanced at lower temperatures. When the stimulation temperature is increased to a certain extent, local oxygen consumption becomes more complex, resulting in a decrease in the correlation between the oxygen saturation signal and blood flow signal.

According to previous studies, there are many frequency bands in the blood flux recorded by LDF^21–24^, which might reflect different physiological rhythms^25^. Kuliga *et al*. analyzed the correlation between oxygen saturation and blood flow signals in the frequency domain, and showed that there is a significant correlation between low-frequency components, such as the endothelial band, the neurogenic band, and the myogenic band^9^. From another perspective, the microcirculatory perfusion can also be distinguished according to the different velocity of blood flow, and the EPOS system can distinguish different velocity components. Therefore, in the present study, we merely distinguish the correlation between different velocity components and oxygen saturation, and do not analyze the blood and oxygen saturation signals in the frequency domain.

Previous results have suggested that the different hemodynamic states can be distinguished by a scale analysis of blood flux^26^. The CAI proposed by Costa *et al*.^18^ was calculated in the current study using the following steps: (1) coarse graining of the oxygen saturation signals to obtain new multiple signals series, each of which acquires the system dynamics at this scale; (2) calculating the fuzzy entropy and SampEn for oxygen saturation; and (3) integrating the values of fuzzy entropy or SampEn within the predefined scale to obtain the CAI^17,27^. Previous studies have shown that the CAI has potential value in the diagnosis and prognosis of specific diseases. Decreased MSE values of Electroencephalogram in patient with neonatal seizures reflect who will be more likely to suffer later epilepsy^28^. MSE complexity index of both glucose dynamics and fetal heart rate decreased in the patient with diabetic^29^ and acidemia^30^, respectively.

Similar to the results of conventional LDF^20^ and speed-resolved blood flux^10^, stimulation at 38 and 40 °C decreased CAI, while stimulation at 42 and 44 °C increased CAI. These results suggest that in the time domain, the response of the various signals induced by different temperature stimuli is different; however, from the point view of complexity, the change in the complexity of various signals is convergent.

Previous study has suggested that there is a positive correlation between absolute changes in oxygen saturation and relative changes in perfusion induced by thermal stimulation^7^. In the present study, Spearman’s correlation coefficient was used to measure the correlation between the time series of speed-resolved blood flux and oxygen saturation, which is a supplement to previous research^7^. However, the results of the present study indicate that the correlation between the two signals is enhanced by stimulation at lower temperatures, while the correlation is significantly reduced by stimulation at higher temperatures. These results suggest that the effect of temperature stimulation on oxygen saturation and blood flow is not a simple linear relationship. With increasing stimulation temperature, the process of local oxygen metabolism tends to be complex.

From the perspective of signal complexity, our results show that there is little correlation between the complexity of oxygen saturation and the complexity of different speed-resolved blood flux signals in the resting state. With increases in stimulation temperature, the changes in correlation between low-velocity perfusion and oxygen saturation became more obvious. With 44 °C stimulation, the correlation even reversed from positive to negative. These data indicate that the relationship between low-velocity components and oxygen saturation in terms of signal complexity is close and complex, which further complements previous studies^7^.

The current study only investigated temperature stimuli from 38 to 44 °C, and did not observe nociceptive thermal stimulation. Furthermore, the temperature intervals in this study were 2 °C, which is much greater than the resolution of the temperature sensor. Therefore, in future research, we should optimize temperature settings to explore the local response caused by temperature stimulation. The local blood flux response induced by thermal stimulation generally consists of two stages: the first stage is the axonal reflex stage, and the second stage is the plateau stage. Because of the different mechanisms of the two stages, we speculate that the correlation between blood flow and oxygen saturation is different. However, owing to the limited sampling rate, the two stages were not analyzed separately here, which is a limitation of the current study.

## Conclusions

Our experimental results and analyses demonstrate that thermal stimulation significantly increases local oxygen saturation and affects different measures of complexity of local oxygen saturation in a temperature-dependent manner. The complexity area index is at first significantly reduced in response to 38 and 40 °C but subsequently increases upon stimulation at higher temperatures. The correlation analysis in the time domain shows that the correlation between oxygen saturation and speed-resolved blood flux increases when stimulated at 38 °C but decreases significantly at higher temperatures. When considering the data from the point of view of complexity, we observed little correlation between the oxygen saturation and speed-resolved blood flux in the resting state before stimulation. Upon increasing the stimulation temperature, a correlation gradually appears, making the change between low-velocity components and oxygen saturation more apparent. The correlation between medium-velocity components and oxygen saturation was found at 38 °C and 42 °C stimulation, while there was a significant correlation between high-velocity components and oxygen saturation at 40 °C stimulation.

## Materials and Methods

### Participants and experimental design

Healthy subjects aged 18 to 60 years were recruited and assigned to one of two categories: “thermal stimulation” (TS, N = 29) and “blank controls” (BC, N = 30). One subject’s oxygen saturation data can not be imported into Matlab software and was discarded from the analysis. As reported earlier^10^, there was no significant difference in age, sex and MBI between the two groups. Alcohol, tea, and coffee intake were prohibited for at least 24 h prior to measurements. None of the subjects were taking any medication that would affect cardiovascular or autonomic regulation.

Measurements were performed between 9 a.m. and 5 p.m. in a quiet, temperature-controlled (24–26 °C) laboratory. After a period of cardiovascular stability (30 min), a baseline recording was made for 30 min for all participants to ensure stabilization. Subsequently, the individuals in the TS group received four stimuli at 38, 40, 42, and 44 °C using an EPOS thermal stimulation unit (Perimed AB, Stockholm, Sweden). Each temperature stimulus lasted for 30 minutes, and then the probe automatically turned off the heating function according to the software settings. And then followed by another 30 minutes recording. The whole recording process was completed by software setting without any manual operation (Fig. 1b). To enable the measurement of blood-flow parameters in the resting state, the BC group underwent the same procedure as the TS group except that the probe was not heated.

### Oxygen saturation measurements and analysis

During recording sessions, subjects were placed in a supine position and their forearms were fixed with a vacuum pillow (AB Germa, Kristianstad, Sweden). A multimodal assessment of microcirculation via diffuse reflectance spectroscopy (DRS) and laser Doppler flowmetry (LDF) was performed by EPOS (Perimed AB, Stockholm, Sweden) with a 3 Hz sampling rate as previously described^5,6^. The system relies on model-based method were model parameters including oxygen saturation, amount and speed-distribution of blood, scattering properties etc., are iteratively updated until modeled and measured spectra agree. The method has been thoroughly described and verified before^1,31,32^. All measurements were set up within the EPOS management system. The probe, which was fixed using double-sided adhesive tape (PF 105-1, Perimed AB, Stockholm, Sweden), combined both a laser Doppler probe and a thermostatic probe at the stimulated point, which allowed for the controlled and consistent heating of the area of the skin under the surface of the probe. The results recorded were exported directly (in MATLAB format) from the EPOS system into MATLAB 2015b (MathWorks, Natick, MA, USA) for analysis. The mean oxygen saturation was averaged over one minute (30 seconds before and 30 seconds after) for each time point.

### Complexity of the oxygen saturation signal

The complexity of the blood flux signal was evaluated by MSE and MFE. The theory behind the effectiveness of MSE in teasing apart the complexity of physiological signals has been previously described by Costa *et al*.^13,14^, while the analytical methods of the MATLAB toolbox were provided by PhysioNet^33^. MFE was calculated using the MATLAB toolbox provided by Azami, H *et al*.^34^. In addition, we calculated the complexity area index for each coarse-grained time series, which was defined as the integral of the sample entropy curve^27,35^. The parameters of the analysis were described in a previous study (for MSE: m = 2, r = 0.15; for MFE: m = 2, r = 0.15, fuzzy power = 2)^20^.

### Statistical analysis

The data are presented as the mean ± SE. Between-subject factors were analyzed using a mixed repeated-measures ANOVA with SPSS software 23.0 (IBM Corp., Armonk, NY, USA), while a permutation test was used for posttests. Multiple comparisons among groups were performed as an independent design. All permutation test analyses were conducted using MATLAB 2015b (MathWorks, Natick, MA, USA). All reported *P* values were two-sided, and significance was defined as *P* < 0.05.

### Ethical approval and consent to participate

This study was approved by the Institutional Research Ethics Boards of Acupuncture & Moxibustion, China Academy of Chinese Medical Sciences. In accordance with the Declaration of Helsinki, each subject provided informed consent and had an adequate understanding of the procedure and purpose of the study.

## Supplementary information

Supplementary information.

## Data Availability

Guangjun, W., Shuyong, J., Xiaojing, S., Weibo, Z. (2019): A dataset of speed-resolved blood perfusion and oxygen saturation after different thermal stimulation. figshare 10.6084/m9.figshare.8299343.v4.
