# Supplementary material for: Impact of local thermal stimulation on the correlation between oxygen saturation and speed-resolved blood perfusion
Source: Sci Rep. 2020 Jan 13;10:183. doi: 10.1038/s41598-019-57067-6 (PMC6957488; doi:10.1038/s41598-019-57067-6)
Supplement: Supplementary file 1 — Supplementary information. [file 41598_2019_57067_MOESM1_ESM.docx]

**Impact of local thermal stimulation on the correlation between oxygen saturation and speed-resolved blood perfusion**

Guangjun Wang^1^*, Shuyong Jia^1^, Mi Liu^2^, Xiaojing Song^1^, Hongyan Li^1^, Xiaorong Chang^2^*, Weibo Zhang^1^*

1. Institute of Acupuncture and Moxibustion, China Academy of Chinese Medical Sciences, Beijing, China
2. Acupuncture and Tuina School, Hunan University of Chinese Medicine, Changsha, China

*Correspondence to tjuwgj@gmail.com; [xrchang1956@163.com](mailto:xrchang1956@163.com); [zhangweibo@hotmail.com](mailto:zhangweibo@hotmail.com)


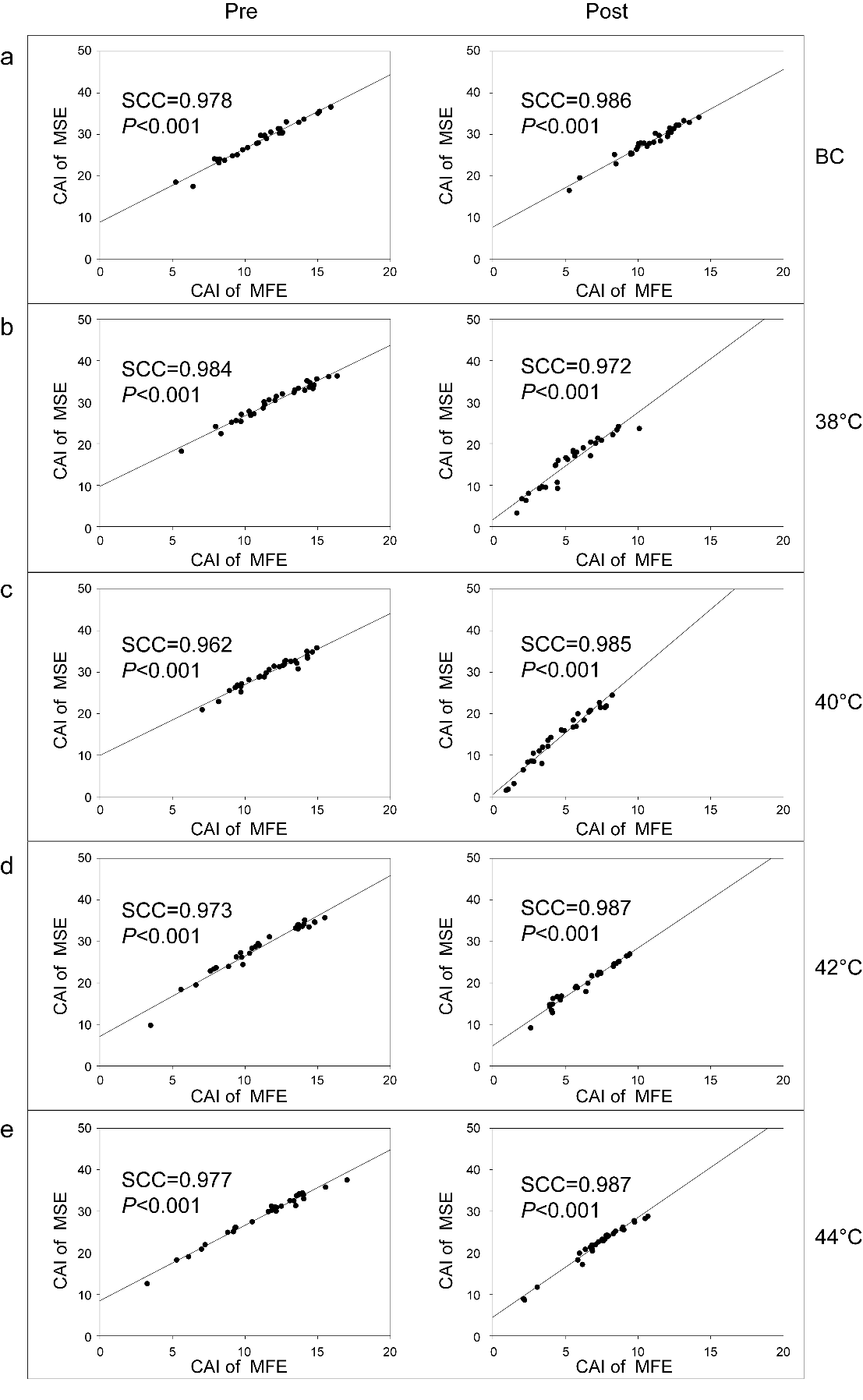


Fig. The relationship of CAI between MFE and MSE. The columns correspond to the Pre and Post stimulation: (a) background control, and thermal stimulation at (b) 38°C, (c) 40°C, (d) 42°C, and (e) 44°C. CAI, complexity area index; SCC, Spearman’s correlation coefficient.


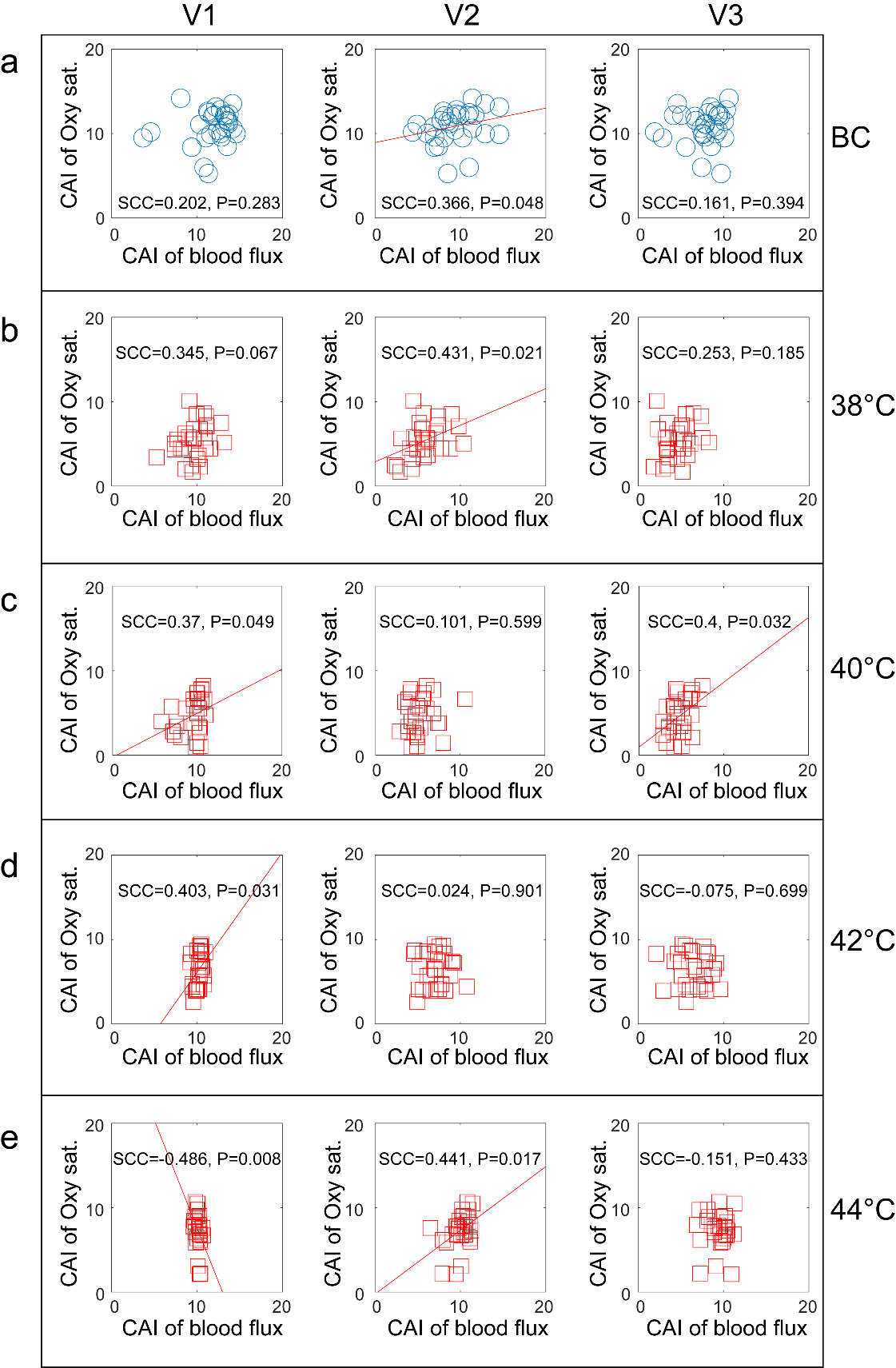


Fig. S2 Spearman correlation between the MFE CAI data for speed-resolved blood perfusion and oxygen saturation signals. The columns correspond to the three velocity components V1, V2, and V3, and the rows to the stimulation protocol: (a) background control, and thermal stimulation at (b) 38°C, (c) 40°C, (d) 42°C, and (e) 44°C. SCC, Spearman’s correlation coefficient; CAI, complexity area index. Oxy sat., oxygen saturation.
